# Crystal structure and Hirshfeld surface analysis of diethyl 2-[4-(4-fluoro­phen­yl)-2-methyl-4-oxobutan-2-yl]malonate

**DOI:** 10.1107/S2056989018012094

**Published:** 2018-09-07

**Authors:** Sandeep Chandrashekharappa, Keshab M. Bairagi, Mahendra K. Mohan, Katharigatta N. Venugopala, Susanta K. Nayak

**Affiliations:** a Institute for Stem Cell Biology and Regenerative Medicine (inStem), GKVK Campus, Bellary Road, Bangalore 560 065, Karnataka, India; b Department of Chemistry, Visvesvaraya National Institute of Technology, Nagpur 440 010, Maharashtra, India; c Department of Biotechnology and Food Technology, Durban University of Technology, Durban 4001, South Africa

**Keywords:** crystal structure, malonate, Hirshfeld surface

## Abstract

The title compound, C_18_H_23_FO_5_, was synthesized by reacting diethyl malonate with 1-(4-fluoro­phen­yl)-3-methyl­but-2-en-1-one. The mol­ecule adopts a loose conformation stabilized by weak C—H⋯O and C—H⋯π inter­actions. In the crystal, the mol­ecules are joined by C—H⋯O contacts into infinite chains along the *b-*axis direction with a *C*(6) graph-set motif. Hirshfeld surface analysis and fingerprint plots demonstrate the predominance of H⋯H, O⋯H and F⋯H inter­molecular inter­actions in the crystal structure.

## Abstract 2

The title compound was synthesized by reacting diethyl malonate with 1-(4-fluoro­phen­yl)-3-methyl­but-2-en-1-one. In the crystal, the mol­ecules are joined by C—H⋯O contacts into infinite chains along the *b-*axis direction with a *C*(6) graph-set motif.

## Chemical context   

Polyfunctionalized reactions are used to synthesize the bioactive compounds that are inter­esting core structures for the development of new drug mol­ecules. The direct functionalization of chemical inter­mediates has attracted extensive attention of synthetic chemists (Fournier *et al.*, 1994 ▸; Liu & Couldwell, 2005 ▸; Markham & Faulds, 1998 ▸) for the construction of heterocyclic compounds that are known to exhibit various pharmacological properties such as anti­cancer (Kasumbwe *et al.*, 2017 ▸), anti­mosquito (Venugopala *et al.*, 2013*a* ▸), anti-tubercular (Narayanaswamy *et al.*, 2013*b* ▸), anti-HIV (Poty *et al.*, 2015 ▸), anti-diabetic (Shahidpour *et al.*, 2015 ▸) and anti-microbial (Ji *et al.*, 2015 ▸) activities. The title compound, achieved by Michael addition (Simamura *et al.*, 1954 ▸), is an important precursor in the construction of the heterocyclic compound *N*2-(3-(di­fluoro­meth­oxy)-4-(3-methyl-1*H*-1,2,4-triazol-1-yl)phen­yl)-7-(4-fluoro­phen­yl)-*N*4,5,5-tri­methyl-6,7-di­hydro-5*H*-cyclo­penta­[*d*]pyrimidine-2,4-di­amine, which is a modulator of β-amyloid peptide production in treating Alzheimer’s disease (Boy *et al.*, 2015 ▸).
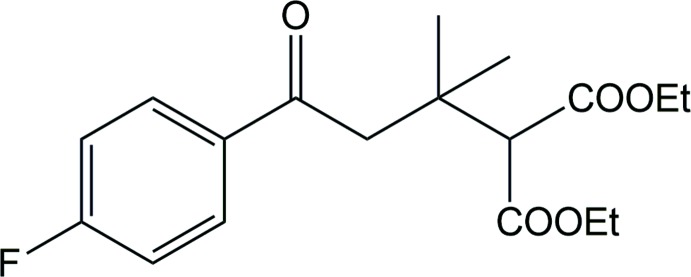


## Structural commentary   

**Figure 1:**
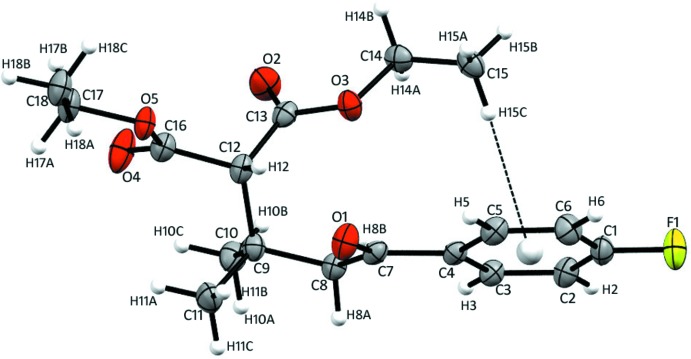
The asymmetric unit of the title compound with 50% probability ellipsoids with atom labelling. The intra­molecular C—H⋯π inter­action is shown as a dotted line.

The title compound crystallizes in the monoclinic crystal system in the space group *P*2_1_/*n*, with one mol­ecule in the asymmetric unit (*Z*′ = 1). The mol­ecular conformation is stabilized by an intra­molecular C—H⋯O hydrogen bonds and C—H⋯π inter­action (Fig. 1 ▸, Table 1 ▸) and short O3⋯O7 contact [3.007 (2) Å]. All bonds between *sp* ^3^-hybridized atoms adopt staggered conformations, thus indicating that steric tensions are absent from this mol­ecule. The dihedral angle between the two ester groups of the malonate residue is 61.79 (5)°; the dihedral angles formed by aromatic ring with adjacent and opposite ester groups are 56.66 (4) and 16.08 (4)°, respectively. The dihedral angle between aromatic ring and ketone carbonyl unit is 14.04 (5)°.

## Supra­molecular features   

**Figure 2:**
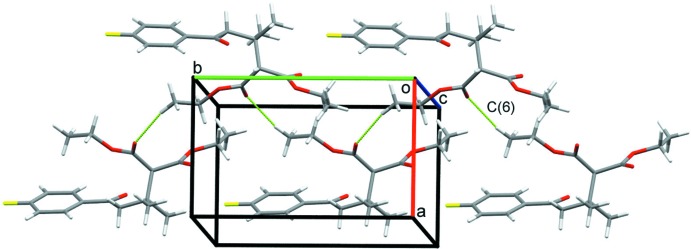
Crystal packing of the title compound. The C—H⋯O hydrogen bonds form infinite chains along the *b*-axis direction.

In the crystal of the title compound, the shortest inter­molecular contact is C15–H15*B*⋯O2, which join the mol­ecules into infinite chains with graph-set motif *C*(6) (Etter *et al.*, 1990 ▸) along the *b*-axis direction (Table 1 ▸, Fig. 2 ▸). There are also a few other H⋯O contacts at the level of the sum of covalent radii.

## Hirshfeld surfaces analysis   

**Figure 3:**
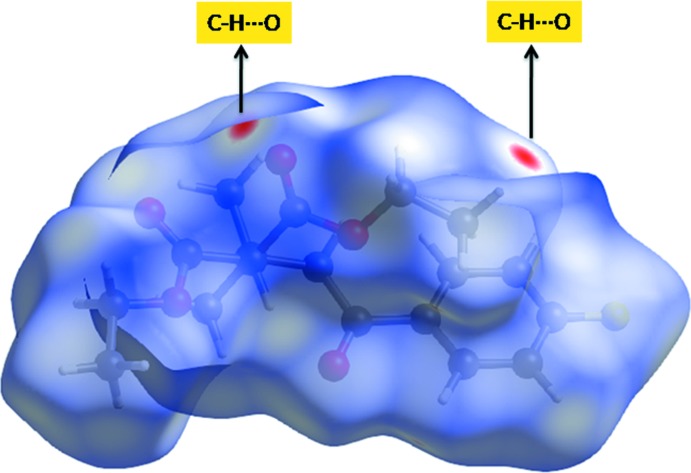
Hirshfeld surface of the title compound mapped over *d*_norm_.

**Figure 4:**
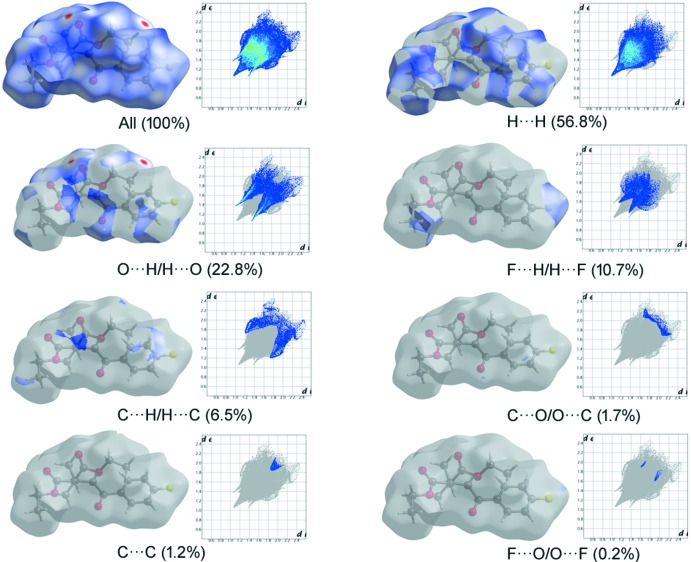
Two-dimensional fingerprint plots and relative contributions of various inter­actions to the Hirshfeld surface of the title compound.

The approach based on Hirshfeld surfaces is a tool for visualizing the inter­molecular inter­action (Spackman & Jayatilaka, 2009 ▸). The Hirshfeld surfaces and two-dimensional fingerprint plot generated using *CrystalExplorer* 3.1 (Wolff *et al.*, 2012 ▸) are presented in Figs. 3 ▸ and 4 ▸. The red spots on the Hirshfeld surface correspond to the C15—H15*B*⋯O2 contact, whereas the blue areas are completely free from close contacts, thus indicating that the only important contact is of the C—H⋯O type. The fingerprint plots (Fig. 4 ▸) show that the H⋯H inter­molecular contacts give the largest contribution of 56.8%, and the observed white spots on the *d*_norm_ surface are considered to be weak inter­actions. The O⋯H/H⋯O contacts, which are shown as a sharp spike in the fingerprint plots, correspond to 22.8% of the total inter­actions. The percentage contribution of other weak inter­actions are as follows: H⋯F/F⋯H – 10.7%, C⋯H/H⋯C – 6.5%, C⋯O/O⋯C – 1.7%, C⋯C – 1.2% and F⋯O/O⋯F – 0.2%.

## Database survey   

A search in the Cambridge Structural Database (version 5.39, last updated May 2018; Groom *et al.*, 2016 ▸) for the fragments F—C_6_H_4_—C(=O)—CH_2_ and C_6_H_4_—C(=O)—CH_2_—CH_2_—CH(COO)_2_ gave 102 and 62 hits, respectively. Among them, two hits, (*S*)-ethyl-2-(4-*t*-butyl­benzyl­sulfan­yl)-4-(4-fluoro­phen­yl)-4-oxo­butano­ate (refcode: YOGMEO; Kowalczyk *et al.*, 2014 ▸) and dimethyl (*S*)-2-(1-(4-nitro­phen­yl)-1,4-dioxo­pentan-3-yl) malonate (refcode: YUFSOJ; Lippur *et al.*, 2015 ▸) are the most closely related to the title crystal structure. The dihedral angles between the adjacent alkyl ester group and the aromatic ring in YOGMEO, YUFSOJ and the title structure are 78.97 (3), 39.37 (2) and 56.66 (4)°, respectively. As in the title structure, in YUFSOJ there are inter­molecular C—H⋯O contacts involving the methyl groups, whereas in YOGMEO the C—H⋯O contacts are formed with a hydrogen atom of the aromatic group.

## Synthesis and crystallization   

To a stirred solution of diethyl malonate (1 g, 6.25 mmol) in tetra­hydro­furan (5 ml), sodium hydride (0.33 g, 13.75 mmol) was added at 273 K. The reaction mixture was allowed to stir for 15 min. A solution of 1-(4-fluoro­phen­yl)-3-methyl­but-2-en-1-one (1.11 g, 6.25 mmol) in THF was added into the reaction mixture. The reaction mixture was then allowed to stir overnight at room temperature. The completion of the reaction was monitored by thin layer chromatography. The reaction mixture was quenched with saturated ammonium chloride and extracted with ethyl acetate (2 × 25 ml). The combined organic layer was washed with water (2 × 25 ml), brine (25 ml), dried over sodium sulfate and evaporated under reduced pressure to obtain the crude product, which was purified by column chromatography using 60–120 mesh silica gel with ethyl acetate and hexane eluent (*v*/*v* = 1:2). Single crystals of the title compound were obtained by slow evaporation from acetone solvent at room temperature.

## Refinement   

Crystal data, data collection and structure refinement details are summarized in Table 2 ▸. All hydrogen atoms were placed in idealized positions (C—H = 0.95–1.00 Å) and refined using riding model with *U*_iso_ = 1.2 or 1.5*U*_eq_(C). The methyl groups were allowed to rotate.

## Supplementary Material

Crystal structure: contains datablock(s) I. DOI: 10.1107/S2056989018012094/yk2116sup1.cif

Structure factors: contains datablock(s) I. DOI: 10.1107/S2056989018012094/yk2116Isup2.hkl

Supporting information file. DOI: 10.1107/S2056989018012094/yk2116Isup3.cml

CCDC reference: 1863914

Additional supporting information:  crystallographic information; 3D view; checkCIF report

## Figures and Tables

**Table 1:** Hydrogen-bond geometry (Å, °) *Cg* is the centroid of the C1–C6 aromatic ring.

*D*—H⋯*A*	*D*—H	H⋯*A*	*D*⋯*A*	*D*—H⋯*A*
C10—H10*C*⋯O4	0.98	2.40	3.057 (2)	124
C11—H11*B*⋯O1	0.98	2.55	3.167 (2)	121
C12—H12⋯O1	1.00	2.36	3.056 (2)	126
C15—H15*B*⋯O2^i^	0.98	2.54	3.500 (2)	168
C15—H15*C*⋯*Cg*	0.98	2.93	3.836 (2)	154

Symmetry code: (i) 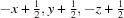.

**Table 2:** Experimental details

Crystal data	
Chemical formula	C_18_H_23_FO_5_
*M*_r_	338.36
Crystal system, space group	Monoclinic, *P*2_1_/*n*
Temperature (K)	153
*a*, *b*, *c* (Å)	7.3066 (6), 11.5182 (9), 20.2701 (17)
β (°)	93.673 (2)
*V* (Å^3^)	1702.4 (2)
*Z*	4
Radiation type	Mo *K*α
μ (mm^−1^)	0.10
Crystal size (mm)	0.22 × 0.13 × 0.10

Data collection	
Diffractometer	Bruker Kappa DUO APEXII
Absorption correction	Multi-scan (*SADABS*; Sheldrick, 2015 ▸)
*T*_min_, *T*_max_	0.929, 0.941
No. of measured, independent and observed [*I* > 2σ(*I*)] reflections	15690, 4045, 2835
*R*_int_	0.053
(sin θ/λ)_max_ (Å^−1^)	0.657

Refinement	
*R*[*F* ^2^ > 2σ(*F* ^2^)], *wR*(*F* ^2^), *S*	0.042, 0.104, 1.03
No. of reflections	4045
No. of parameters	222
H-atom treatment	H-atom parameters constrained
Δρ_max_, Δρ_min_ (e Å^−3^)	0.26, −0.21

Computer programs: *APEX2* and *SAINT* (Bruker, 2014 ▸), *SHELXS97* (Sheldrick, 2008 ▸), *SHELXL2014* (Sheldrick, 2015 ▸), *PLATON* (Spek, 2009 ▸), *Mercury* (Macrae *et al.*, 2008 ▸), *WinGX* (Farrugia, 2012 ▸) and *PARST* (Nardelli, 1995 ▸).

## supplementary crystallographic information

### Crystal data

**Table:**

C_18_H_23_FO_5_	*F*(000) = 720
*M**_r_* = 338.36	*D*_x_ = 1.320 Mg m^−^^3^*D*_m_ = 1.32 Mg m^−^^3^*D*_m_ measured by
Monoclinic, *P*2_1_/*n*	Mo *K*α radiation, λ = 0.71073 Å
*a* = 7.3066 (6) Å	Cell parameters from 2274 reflections
*b* = 11.5182 (9) Å	θ = 5.4–52.6°
*c* = 20.2701 (17) Å	µ = 0.10 mm^−^^1^
β = 93.673 (2)°	*T* = 153 K
*V* = 1702.4 (2) Å^3^	Block, colorless
*Z* = 4	0.22 × 0.13 × 0.10 mm

### Data collection

**Table:**

Bruker Kappa DUO APEXII diffractometer	2835 reflections with *I* > 2σ(*I*)
Radiation source: fine-focus sealed tube	*R*_int_ = 0.053
0.5° φ scans and ω scans	θ_max_ = 27.8°, θ_min_ = 2.0°
Absorption correction: multi-scan (SADABS; Sheldrick, 2015)	*h* = −9→9
*T*_min_ = 0.929, *T*_max_ = 0.941	*k* = −15→15
15690 measured reflections	*l* = −26→23
4045 independent reflections	

### Refinement

**Table:**

Refinement on *F*^2^	Secondary atom site location: difference Fourier map
Least-squares matrix: full	Hydrogen site location: inferred from neighbouring sites
*R*[*F*^2^ > 2σ(*F*^2^)] = 0.042	H-atom parameters constrained
*wR*(*F*^2^) = 0.104	*w* = 1/[σ^2^(*F*_o_^2^) + (0.0423*P*)^2^ + 0.1543*P*] where *P* = (*F*_o_^2^ + 2*F*_c_^2^)/3
*S* = 1.02	(Δ/σ)_max_ < 0.001
4045 reflections	Δρ_max_ = 0.26 e Å^−^^3^
222 parameters	Δρ_min_ = −0.21 e Å^−^^3^
0 restraints	Extinction correction: SHELXL2014 (Sheldrick, 2015), Fc^*^=kFc[1+0.001xFc^2^λ^3^/sin(2θ)]^-1/4^
Primary atom site location: structure-invariant direct methods	Extinction coefficient: 0.0022 (6)

### Special details

**Table:**

Geometry. All esds (except the esd in the dihedral angle between two l.s. planes) are estimated using the full covariance matrix. The cell esds are taken into account individually in the estimation of esds in distances, angles and torsion angles; correlations between esds in cell parameters are only used when they are defined by crystal symmetry. An approximate (isotropic) treatment of cell esds is used for estimating esds involving l.s. planes.

### Fractional atomic coordinates and isotropic or equivalent isotropic displacement parameters (Å^2^)

**Table:**

	*x*	*y*	*z*	*U*_iso_*/*U*_eq_	
F1	0.81620 (15)	0.89772 (7)	0.46296 (5)	0.0405 (3)	
O1	0.74992 (16)	0.35297 (9)	0.46976 (5)	0.0307 (3)	
O2	0.42377 (17)	0.28973 (10)	0.24940 (5)	0.0383 (3)	
O3	0.45134 (15)	0.40181 (8)	0.34014 (5)	0.0262 (3)	
O4	0.56685 (18)	0.04282 (10)	0.28153 (5)	0.0411 (3)	
O5	0.45277 (15)	0.05130 (8)	0.38152 (5)	0.0258 (3)	
C1	0.8140 (2)	0.78062 (13)	0.45372 (7)	0.0268 (4)	
C2	0.8913 (2)	0.73723 (13)	0.39876 (7)	0.0260 (3)	
H2	0.9456	0.7873	0.3683	0.031*	
C3	0.8875 (2)	0.61769 (12)	0.38918 (7)	0.0221 (3)	
H3	0.9381	0.5856	0.3512	0.026*	
C4	0.8102 (2)	0.54422 (12)	0.43457 (7)	0.0205 (3)	
C5	0.7325 (2)	0.59304 (13)	0.48947 (7)	0.0247 (3)	
H5	0.6782	0.5440	0.5204	0.030*	
C6	0.7340 (2)	0.71182 (13)	0.49918 (7)	0.0285 (4)	
H6	0.6809	0.7451	0.5364	0.034*	
C7	0.8023 (2)	0.41483 (13)	0.42581 (7)	0.0217 (3)	
C8	0.8624 (2)	0.36640 (12)	0.36098 (7)	0.0224 (3)	
H8A	0.9977	0.3724	0.3620	0.027*	
H8B	0.8129	0.4183	0.3252	0.027*	
C9	0.8105 (2)	0.24080 (12)	0.34080 (7)	0.0223 (3)	
C10	0.8648 (2)	0.22647 (14)	0.26926 (7)	0.0294 (4)	
H10A	0.9970	0.2395	0.2675	0.044*	
H10B	0.7978	0.2830	0.2408	0.044*	
H10C	0.8344	0.1477	0.2538	0.044*	
C11	0.9171 (2)	0.15246 (13)	0.38520 (8)	0.0331 (4)	
H11A	0.8871	0.0736	0.3699	0.050*	
H11B	0.8832	0.1616	0.4309	0.050*	
H11C	1.0491	0.1659	0.3831	0.050*	
C12	0.5999 (2)	0.22011 (12)	0.34749 (7)	0.0218 (3)	
H12	0.5754	0.2345	0.3948	0.026*	
C13	0.4813 (2)	0.30483 (13)	0.30589 (7)	0.0232 (3)	
C14	0.3506 (2)	0.49428 (13)	0.30408 (7)	0.0261 (4)	
H14A	0.2225	0.4698	0.2930	0.031*	
H14B	0.4089	0.5117	0.2624	0.031*	
C15	0.3541 (2)	0.60000 (13)	0.34758 (8)	0.0288 (4)	
H15A	0.2984	0.5814	0.3890	0.043*	
H15B	0.2848	0.6627	0.3249	0.043*	
H15C	0.4812	0.6248	0.3572	0.043*	
C16	0.5405 (2)	0.09577 (13)	0.33143 (7)	0.0247 (3)	
C17	0.3988 (2)	−0.07089 (12)	0.37518 (7)	0.0285 (4)	
H17A	0.5063	−0.1196	0.3666	0.034*	
H17B	0.3050	−0.0809	0.3382	0.034*	
C18	0.3222 (2)	−0.10521 (13)	0.43942 (8)	0.0325 (4)	
H18A	0.4151	−0.0925	0.4757	0.049*	
H18B	0.2879	−0.1875	0.4378	0.049*	
H18C	0.2137	−0.0580	0.4466	0.049*	

### Atomic displacement parameters (Å^2^)

**Table:**

	*U*^11^	*U*^22^	*U*^33^	*U*^12^	*U*^13^	*U*^23^
F1	0.0591 (7)	0.0204 (5)	0.0428 (6)	0.0001 (4)	0.0096 (5)	−0.0073 (4)
O1	0.0479 (8)	0.0261 (6)	0.0187 (5)	−0.0084 (5)	0.0049 (5)	0.0033 (5)
O2	0.0477 (8)	0.0406 (7)	0.0252 (6)	0.0070 (6)	−0.0085 (5)	−0.0085 (5)
O3	0.0358 (7)	0.0219 (6)	0.0208 (5)	0.0039 (5)	0.0003 (4)	0.0000 (4)
O4	0.0665 (9)	0.0319 (7)	0.0269 (6)	−0.0175 (6)	0.0188 (6)	−0.0121 (5)
O5	0.0378 (7)	0.0175 (5)	0.0227 (5)	−0.0069 (5)	0.0075 (5)	−0.0008 (4)
C1	0.0324 (9)	0.0199 (8)	0.0279 (8)	−0.0001 (6)	−0.0004 (7)	−0.0038 (6)
C2	0.0322 (9)	0.0245 (8)	0.0216 (8)	−0.0043 (6)	0.0038 (6)	0.0014 (6)
C3	0.0236 (8)	0.0250 (8)	0.0178 (7)	−0.0026 (6)	0.0038 (6)	−0.0028 (6)
C4	0.0222 (8)	0.0220 (7)	0.0172 (7)	−0.0023 (6)	−0.0010 (6)	−0.0002 (6)
C5	0.0281 (9)	0.0281 (8)	0.0182 (7)	−0.0042 (6)	0.0031 (6)	−0.0004 (6)
C6	0.0333 (9)	0.0322 (9)	0.0208 (8)	0.0015 (7)	0.0069 (6)	−0.0070 (7)
C7	0.0224 (8)	0.0238 (8)	0.0187 (7)	−0.0034 (6)	−0.0009 (6)	0.0005 (6)
C8	0.0262 (8)	0.0219 (8)	0.0194 (7)	−0.0025 (6)	0.0041 (6)	0.0001 (6)
C9	0.0270 (8)	0.0197 (8)	0.0206 (7)	−0.0008 (6)	0.0048 (6)	−0.0012 (6)
C10	0.0324 (9)	0.0297 (9)	0.0273 (8)	−0.0027 (7)	0.0105 (7)	−0.0068 (7)
C11	0.0372 (10)	0.0239 (8)	0.0376 (9)	0.0034 (7)	−0.0032 (7)	0.0006 (7)
C12	0.0299 (9)	0.0196 (7)	0.0162 (7)	−0.0042 (6)	0.0049 (6)	−0.0007 (6)
C13	0.0244 (8)	0.0244 (8)	0.0213 (8)	−0.0054 (6)	0.0056 (6)	−0.0011 (6)
C14	0.0276 (9)	0.0279 (8)	0.0227 (8)	0.0013 (6)	0.0004 (6)	0.0070 (6)
C15	0.0320 (9)	0.0273 (9)	0.0275 (8)	0.0058 (7)	0.0048 (7)	0.0055 (7)
C16	0.0295 (9)	0.0234 (8)	0.0214 (8)	−0.0037 (6)	0.0036 (6)	−0.0009 (6)
C17	0.0412 (10)	0.0177 (8)	0.0266 (8)	−0.0071 (7)	0.0031 (7)	−0.0024 (6)
C18	0.0450 (11)	0.0229 (8)	0.0302 (9)	−0.0092 (7)	0.0073 (7)	−0.0006 (7)

### Geometric parameters (Å, º)

**Table:**

F1—C1	1.3617 (17)	C9—C11	1.537 (2)
O1—C7	1.2212 (17)	C9—C12	1.572 (2)
O2—C13	1.2070 (17)	C10—H10A	0.9800
O3—C13	1.3407 (17)	C10—H10B	0.9800
O3—C14	1.4633 (17)	C10—H10C	0.9800
O4—C16	1.2070 (17)	C11—H11A	0.9800
O5—C16	1.3372 (17)	C11—H11B	0.9800
O5—C17	1.4650 (17)	C11—H11C	0.9800
C1—C6	1.374 (2)	C12—C13	1.523 (2)
C1—C2	1.375 (2)	C12—C16	1.5254 (19)
C2—C3	1.391 (2)	C12—H12	1.0000
C2—H2	0.9500	C14—C15	1.503 (2)
C3—C4	1.396 (2)	C14—H14A	0.9900
C3—H3	0.9500	C14—H14B	0.9900
C4—C5	1.399 (2)	C15—H15A	0.9800
C4—C7	1.502 (2)	C15—H15B	0.9800
C5—C6	1.382 (2)	C15—H15C	0.9800
C5—H5	0.9500	C17—C18	1.503 (2)
C6—H6	0.9500	C17—H17A	0.9900
C7—C8	1.518 (2)	C17—H17B	0.9900
C8—C9	1.5442 (19)	C18—H18A	0.9800
C8—H8A	0.9900	C18—H18B	0.9800
C8—H8B	0.9900	C18—H18C	0.9800
C9—C10	1.537 (2)		
			
C13—O3—C14	116.19 (11)	C9—C11—H11B	109.5
C16—O5—C17	116.16 (11)	H11A—C11—H11B	109.5
F1—C1—C6	118.74 (14)	C9—C11—H11C	109.5
F1—C1—C2	118.01 (14)	H11A—C11—H11C	109.5
C6—C1—C2	123.25 (14)	H11B—C11—H11C	109.5
C1—C2—C3	117.86 (14)	C13—C12—C16	109.85 (12)
C1—C2—H2	121.1	C13—C12—C9	112.35 (12)
C3—C2—H2	121.1	C16—C12—C9	112.98 (12)
C2—C3—C4	120.92 (13)	C13—C12—H12	107.1
C2—C3—H3	119.5	C16—C12—H12	107.1
C4—C3—H3	119.5	C9—C12—H12	107.1
C3—C4—C5	118.84 (13)	O2—C13—O3	123.55 (14)
C3—C4—C7	122.56 (13)	O2—C13—C12	125.75 (14)
C5—C4—C7	118.58 (13)	O3—C13—C12	110.68 (11)
C6—C5—C4	120.75 (14)	O3—C14—C15	107.93 (11)
C6—C5—H5	119.6	O3—C14—H14A	110.1
C4—C5—H5	119.6	C15—C14—H14A	110.1
C1—C6—C5	118.37 (14)	O3—C14—H14B	110.1
C1—C6—H6	120.8	C15—C14—H14B	110.1
C5—C6—H6	120.8	H14A—C14—H14B	108.4
O1—C7—C4	120.29 (13)	C14—C15—H15A	109.5
O1—C7—C8	122.59 (13)	C14—C15—H15B	109.5
C4—C7—C8	117.12 (12)	H15A—C15—H15B	109.5
C7—C8—C9	119.59 (12)	C14—C15—H15C	109.5
C7—C8—H8A	107.4	H15A—C15—H15C	109.5
C9—C8—H8A	107.4	H15B—C15—H15C	109.5
C7—C8—H8B	107.4	O4—C16—O5	123.54 (13)
C9—C8—H8B	107.4	O4—C16—C12	126.51 (14)
H8A—C8—H8B	107.0	O5—C16—C12	109.95 (12)
C10—C9—C11	109.24 (13)	O5—C17—C18	106.84 (12)
C10—C9—C8	106.06 (12)	O5—C17—H17A	110.4
C11—C9—C8	110.99 (12)	C18—C17—H17A	110.4
C10—C9—C12	112.26 (12)	O5—C17—H17B	110.4
C11—C9—C12	108.15 (12)	C18—C17—H17B	110.4
C8—C9—C12	110.15 (12)	H17A—C17—H17B	108.6
C9—C10—H10A	109.5	C17—C18—H18A	109.5
C9—C10—H10B	109.5	C17—C18—H18B	109.5
H10A—C10—H10B	109.5	H18A—C18—H18B	109.5
C9—C10—H10C	109.5	C17—C18—H18C	109.5
H10A—C10—H10C	109.5	H18A—C18—H18C	109.5
H10B—C10—H10C	109.5	H18B—C18—H18C	109.5
C9—C11—H11A	109.5		
			
F1—C1—C2—C3	−179.48 (13)	C11—C9—C12—C13	179.22 (12)
C6—C1—C2—C3	0.1 (2)	C8—C9—C12—C13	57.76 (15)
C1—C2—C3—C4	−1.1 (2)	C10—C9—C12—C16	64.78 (15)
C2—C3—C4—C5	1.4 (2)	C11—C9—C12—C16	−55.81 (15)
C2—C3—C4—C7	179.59 (13)	C8—C9—C12—C16	−177.27 (11)
C3—C4—C5—C6	−0.8 (2)	C14—O3—C13—O2	−3.2 (2)
C7—C4—C5—C6	−179.03 (13)	C14—O3—C13—C12	175.51 (12)
F1—C1—C6—C5	−179.90 (13)	C16—C12—C13—O2	−36.6 (2)
C2—C1—C6—C5	0.6 (2)	C9—C12—C13—O2	90.11 (18)
C4—C5—C6—C1	−0.2 (2)	C16—C12—C13—O3	144.81 (13)
C3—C4—C7—O1	172.84 (14)	C9—C12—C13—O3	−88.52 (15)
C5—C4—C7—O1	−9.0 (2)	C13—O3—C14—C15	−172.32 (13)
C3—C4—C7—C8	−6.9 (2)	C17—O5—C16—O4	4.1 (2)
C5—C4—C7—C8	171.30 (13)	C17—O5—C16—C12	−175.80 (12)
O1—C7—C8—C9	16.5 (2)	C13—C12—C16—O4	72.5 (2)
C4—C7—C8—C9	−163.76 (13)	C9—C12—C16—O4	−53.8 (2)
C7—C8—C9—C10	171.38 (13)	C13—C12—C16—O5	−107.55 (14)
C7—C8—C9—C11	−70.08 (17)	C9—C12—C16—O5	126.14 (13)
C7—C8—C9—C12	49.67 (17)	C16—O5—C17—C18	173.97 (13)
C10—C9—C12—C13	−60.19 (16)		

### Hydrogen-bond geometry (Å, º)

Cg is the centroid of the C1–C6 aromatic ring.

**Table:**

*D*—H···*A*	*D*—H	H···*A*	*D*···*A*	*D*—H···*A*
C10—H10*C*···O4	0.98	2.40	3.057 (2)	124
C11—H11*B*···O1	0.98	2.55	3.167 (2)	121
C12—H12···O1	1.00	2.36	3.056 (2)	126
C15—H15*B*···O2^i^	0.98	2.54	3.500 (2)	168
C15—H15*C*···*Cg*	0.98	2.93	3.836 (2)	154

Symmetry code: (i) −*x*+1/2, *y*+1/2, −*z*+1/2.
